# Identification of novel meQTLs strongly associated with rheumatoid arthritis by large‐scale epigenome‐wide analysis

**DOI:** 10.1002/2211-5463.13517

**Published:** 2022-11-19

**Authors:** Guoping Tang, Chen Sun, Hongchao Lv, Mingming Zhang, Yongshuai Jiang, Jing Xu

**Affiliations:** ^1^ The Fourth Affiliated Hospital Zhejiang University School of Medicine China; ^2^ College of Bioinformatics Science and Technology Harbin Medical University China

**Keywords:** bioinformatics, DNA methylation, epigenetics, genetic risk, rheumatoid arthritis, SNP

## Abstract

Rheumatoid arthritis (RA) is highly heritable, and previous studies have suggested that genetic variation may affect susceptibility to RA by altering epigenetic modifications (e.g. DNA methylation). Here we examined how genetic variation influences DNA methylation (DNAm) in RA by integrating individual genetic variation and DNAm data. Epigenome‐wide meQTL (methylation quantitative trait loci) analysis was performed on 354 RA patients and 335 controls, scanning 30,101,744 relationships between 62 SNPs and 485,512 DNA methylation sites. Two regulatory relationship pairs (FDR < 0.05) showed very strong associations with RA risk. One was rs10796216‐cg00475509, and the DNAm decreased by 0.0168 per addition of allele rs10796216‐A. The other was rs6546473‐cg13358873, for which a 0.0365 reduction of DNAm at cg13358873 was observed for each addition of allele rs6546473‐A, and lower DNAm was found to be significantly associated with RA risk (*P* = 2.0407e‐28). Moreover, both pairs of meQTL showed a strong regulatory relationship only in RA samples, so they can be subsequently considered as risk markers for RA. In conclusion, our integrated analysis of genetic and epigenetic variation suggests that genetic variation may affect the risk of RA by regulating DNA methylation levels. Alterations of DNAm at cg00475509 and cg13358873 loci conferred by rs10796216‐A and rs6546473‐A allele may suggest a potential risk for RA. Our results deepen our understanding of the genetic and epigenetic mechanisms of RA and provide novel associations that can be further investigated in future studies.

## Graphical abstract

Here we performed large‐scale epigenome‐wide meQTL (methylation quantitative trait loci) analysis on 354 RA patients and 335 controls and identified 65 novel meQTLs (FDR < 0.05), among which 21 meQTLs have significant regulatory relationships only in RA samples. Specifically, alterations of DNAm at cg00475509 and cg13358873 conferred by rs10796216‐A and rs6546473‐A showed very strong associations with RA risk.

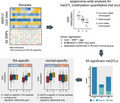

**Abbreviations**
DNAm: DNA methylation
HWE: Hardy–Weinberg equilibrium
MAF: minor allele frequencies
meQTL: methylation quantitative trait loci
RA: rheumatoid arthritis
SNP: single nucleotide polymorphism

Rheumatoid arthritis (RA) is a chronic autoimmune disease [1] that even leads to physical disability at worst. RA patients suffer from long‐term torture but there is still no cure. Studies have shown that RA is highly heritable, with heritability more than 60% [2]. It is necessary to prevent RA at an early stage and reduce the subsequent disease burden, and this can be achieved by predicting the disease susceptibility to RA of individuals in advance based on an individual's genetic information. Large‐scale GWAS studies and function analyses have identified many genetic variants [3] and risk genes [4] contributing to RA susceptibility [5, 6, 7, 8], most of which are more likely in the *HLA* gene region [2, 9]. In addition to genetic factors, RA is also affected by environmental factors.

DNA methylation is a key epigenetic modification, which reflects the changes on individuals brought about by the influence of the environment, to a certain extent. Epigenetics has been proved to have a potential impact on the genetic risk of RA [10, 11] and there may be interactions between genetic variations and epigenetics [12]. This suggests that epigenetics may influence the mechanism of disease, especially through changes caused by genetic variation [13]. However, there is little known about the potential mechanism with RA. Methylation quantitative trait loci (meQTLs) analysis is a common integrative approach for discovering genetic and epigenetic associations. The identification of meQTLs can deepen the understanding of the potential impact of epigenetics in disease genetic mechanisms [14].

Here we aimed to investigate the potential RA pathogenesis‐associated DNA methylation changes that depend on the genotype of genetic variants. We scanned the associations between methylation levels at 485,512 CpG sites and genotypes of 62 SNPs previously unstudied. We finally found 74 significantly associated meQTLs, 27 of which were significantly detected only in RA patients, 15 only significantly present in the normal samples. We then delved into these special regulatory relationships and elucidated them at the functional level.

## Materials and methods

### Datasets

The DNA methylation level datasets were downloaded from the Gene Expression Omnibus (https://www.ncbi.nlm.nih.gov/geo/query/acc.cgi?acc=GSE42861), with accession number GSE42861. The datasets including Illumina HumanMethylation450 arrays on 689 Swedish samples (335 healthy individuals and 354 RA patients) were used to study the potential impact of epigenetic inheritance on the genetic risk of RA.

The SNP genotype data of the 689 individuals was also derived from GSE42861 datasets. In addition to DNA methylation loci, there are 65 SNP probes designed on the Illumina HumanMethylation450 arrays for sample tracking and sample mixups, 62 of which are biallelic SNPs. These SNPs are barely noticed and haven not been studied before. In this study, we aimed to find some new neglected meQTL associations between these SNPs and DNA methylation. The genotypes of these 62 biallelic SNPs were retrieved according to the three distinct peaks of the SNP probe signal that corresponded to homozygotes and heterozygotes. For subsequent analysis, we only include SNPs meeting the following conditions: (a) the minor allele frequencies (MAF) > 0.01, (b) missing rate < 0.01, (c) *P*‐value for Hardy–Weinberg equilibrium (HWE) > 0.001. Finally, all the 62 SNPs passed quality control. This process was performed using the epigenetic association study software ewas2.0 developed previously [15].

### Identification of meQTLs

The meQTL analysis in this study was completed by the software ewas2.0 developed earlier. First, the software encodes the genotype data of the 62 SNPs: if the minor allele is A and the non‐minor allele is a, then the minor allele homozygote “AA” was coded as 2, the heterozygote “Aa” or “aA” was coded as 1, and the nonminor allele homozygote “aa” was coded as 0. Then the relationships between each SNP and all the 485,512 DNA methylation sites were fully scanned, and linear regression analysis with age as the covariate was used to calculate the relationship strength and statistical significance of each SNP‐CpG pair. The DNA methylation level was referred to as the dependent variance and the SNP genotype was referred to as the independent variable. Finally, for each SNP the SNP‐CpG pairs with FDR < 0.05 were screened as significantly correlated meQTL relationship pairs.

### Annotation of meQTLs

Considering that the regulatory relationship between SNPs and CpGs may be different due to their physical location distance on the chromosome, we classified the significantly related meQTL pairs obtained from the screening according to the following rules: (a) if they were located on the same chromosome and the physical location distance was less than 1 Mb, the relationship was considered cis‐regulated (cis meQTL); (b) if they were located on the same chromosome but the physical distance was greater than 1 Mb, the relationship was considered to be long distance ci regulated relationship (long cis meQTL); (c) if they were located on different chromosomes, the relationship was considered to be a transregulated relationship (trans meQTL).

## Results

### Summary of meQTLs

**Fig. 1:**
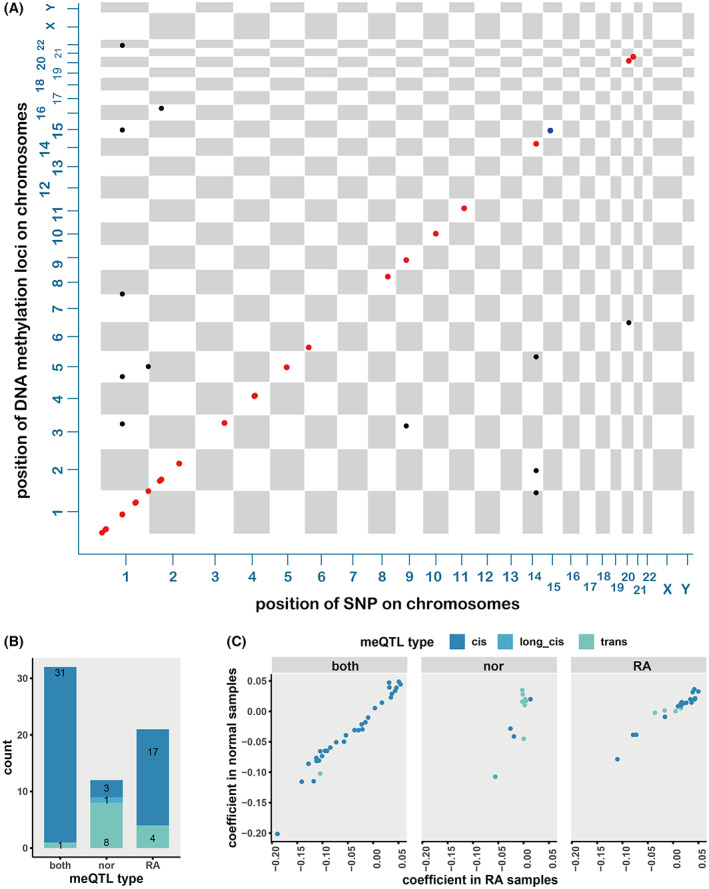
Summary for all meQTLs. (A) Chessboard plot for all meQTLs identified. Each point is an SNP‐CpG pair. The axis‐*x* and axis‐*y* show the location of SNP and CpG on the genome. Points are colored according to the distance between SNP and CpG, red for cis meQTLs, blue for long cis meQTLs, and black for trans meQTLs. (B) The number of meQTLs of each type detected in normal, RA or both. (C) The regression coefficient calculated in RA and normal samples for each meQTL that significantly identified both in normal samples and RA samples (left panel), only in normal samples (middle panel), only in RA samples (right panel).

The DNA methylation data are Illumina HumanMethylation450 arrays data, including 354 RA samples and 335 normal samples in total. RA samples and normal samples were studied separately. Linear regression with age as a covariate was used to scan all the 30,101,744 pair of relationships between 62 SNP sites and 485,512 DNA methylation sites. For each SNP, we selected an epigenome‐wide significance level threshold of FDR < 0.05 to screen meQTL pairs. Finally, we identified 53 SNP‐CpG pairs in RA samples (composing of 21 SNPs and 53 CpG sites) and 44 SNP‐CpG pairs in normal samples (comprised of 17 SNPs and 44 CpG sites) with a significant meQTL effect. The union set of them was selected as discovery meQTL sets, including 65 meQTL that comprisrf 23 SNPs and 65 CpG sites (Fig. 1A).

As the underlying genomic regulatory mechanisms may be different due to the distance between the SNP and CpG sites, we divided the identified meQTLs into cis meQTL, long‐distance cis meQTL, and trans meQTL (see Materials and methods). Among the 64 significant regulatory associations, 51 pairs were cis meQTLs (comprisrf of 22 unique SNPs significantly associated with 51 unique CpG sites); one was long distance cis meQTL, and 13 pairs were trans meQTLs (comprised of six unique SNPs and 13 unique CpG sites) (Fig. 1A).

Some regulatory associations between SNPs and CpG sites were found only significantly in RA patients but not in normal samples. Conversely, some relationships were only detected in normal samples, which suggested that the change of regulatory relationships may be the associated with the RA mechanism. There were 21 regulatory associations (17 cis meQTLs and 4 trans meQTLs) that were significantly detected uniquely in RA samples, 12 unique in normal samples (three cis meQTLs, one long‐distance cis meQTL and eight trans meQTLs), and 31 meQTLs (30 cis meQTLs and one trans meQTLs), both in RA and normal samples (Fig. 1B,C).

**Fig. 2:**
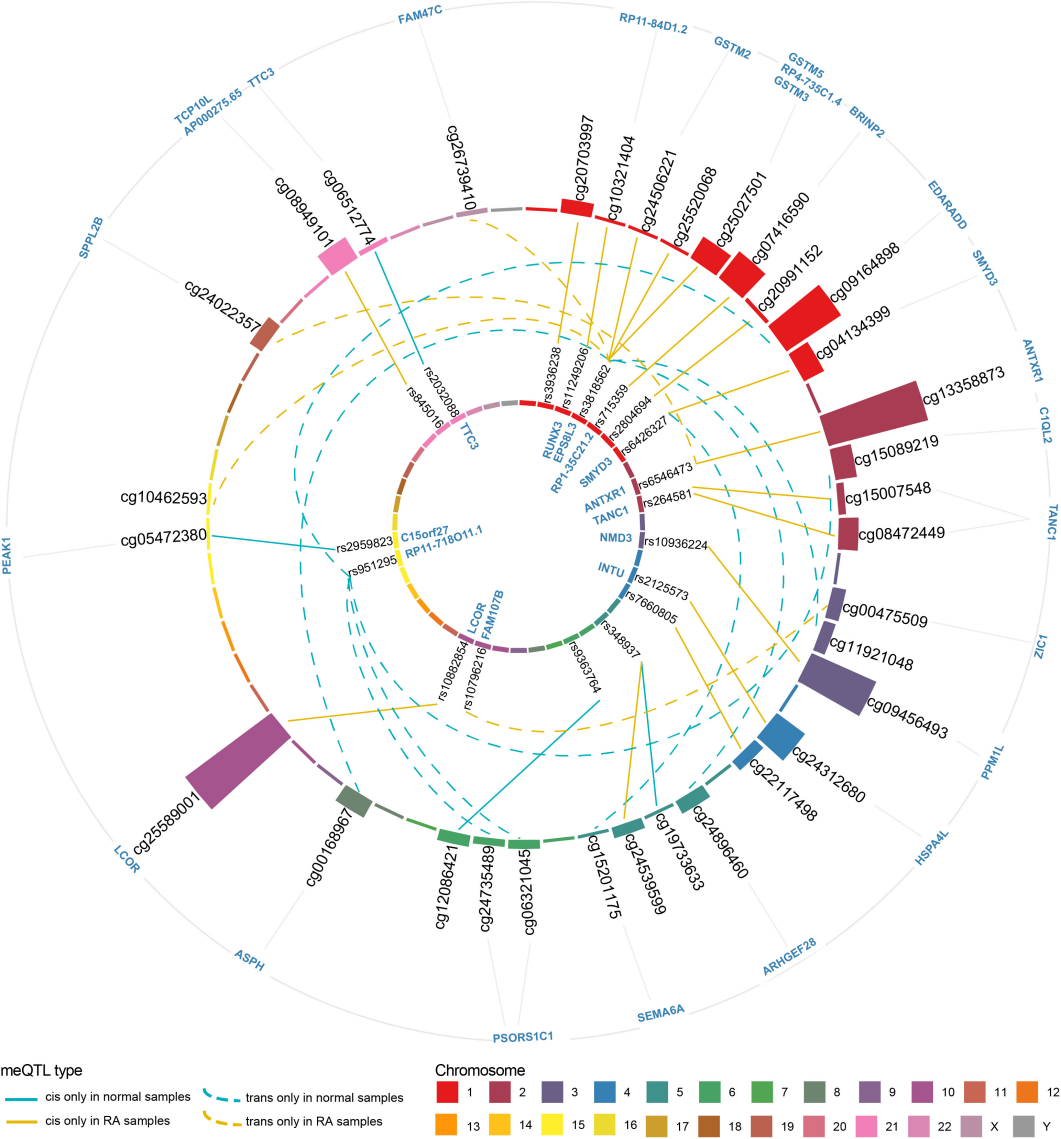
meQTLs identified only in normal samples or RA samples. The inner circle and the outer circle are respectively the SNP site and the CpG site colored according to the chromosome. The height of the column of the outer circle is proportional to the negative logarithm of the *T*‐test *P*‐value for CpG sites. The connecting lines within the two circles represent SNP‐CpG relationship pairs, and each line is colored according to the type of meQTL. The solid/dashed blue line represents the cis/trans meQTL identified only in normal samples, and the solid/dashed yellow line represents the cis/trans meQTL identified only in the RA samples.

In total we found 33 regulatory relationships that were unique in RA or normal samples; for further investigation, involved with 33 DNA methylation sites associated with 19 unique SNPs (Fig. 2).

### Regulatory relationships unique in RA

Here, 17 cis meQTLs and 4 trans meQTLs that have significant regulatory relationships only in the RA sample were obtained (Fig. 2). These meQTLs are involved with 21 CpG sites that are regulated by 15 unique SNPs. There are five meQTL pairs that were located at the same gene, including rs6546473‐cg13358873 pair at gene *ANTXR1* (ANTXR Cell Adhesion Molecule 1), rs10882854‐cg25589001 pair at gene *LCOR*, rs6426327‐cg04134399 pair at gene *SMYD3*, rs264581‐cg08472449 pair, and rs264581‐cg15007548 pair at gene *TANC1*. The *EPS8L3* (EPS8 Like 3) gene employed five meQTLs, followed by *ANTXR1* and *TANC1*, which have two meQTLs. There were four pairs of meQTLs not located on the gene but located in the intergenic region.

**Fig. 3:**
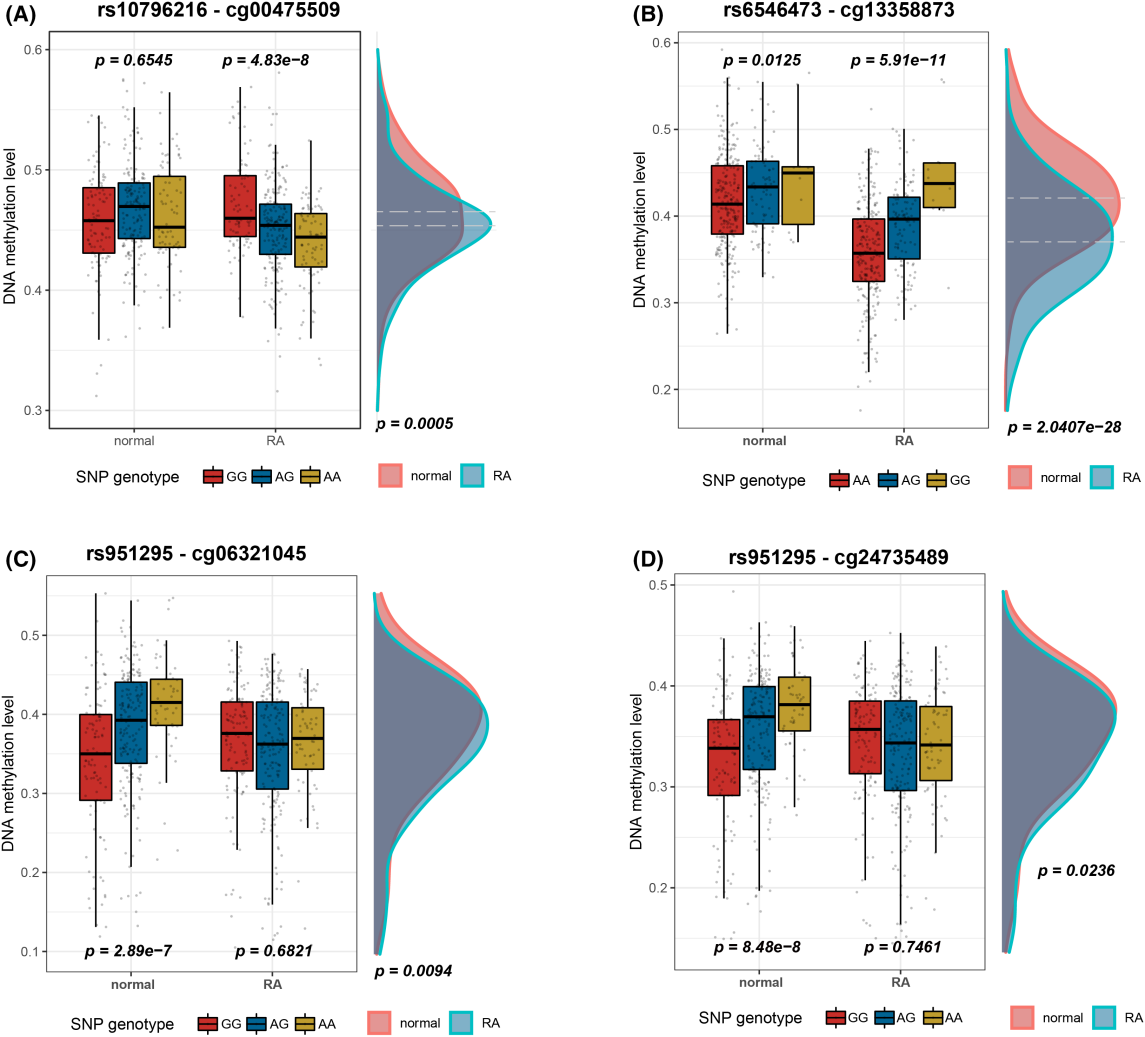
Examples of meQTLs identified only in RA or normal samples. Boxplot of (A) rs10796216‐cg00475509, (B) rs6546473‐cg13358873, (C) rs951295‐cg06321045, and (D) rs951295‐cg24735489 relationship pairs. Relationships between SNP genotypes and DNA methylation level were tested by linear regression, with age included as a covariate, and the difference of DNA methylation level was identified by *t*‐test.

Among the regulatory associations significant only in RA samples, two regulatory relationships were strongly associated with RA risk, including rs10796216‐cg00475509 pair and rs6546473‐cg13358873 pair (Fig. 3A,B).

### Strong regulatory relationships between rs10796216 and cg00475509

The rs10796216‐cg00475509 regulatory relationship pair is a trans meQTL. rs10796216 located in the noncoding area of the *FAM107B* gene on chromosome 10 with two alleles, A and G. cg00475509, located in the *ZIC1* region on chromosome 3. There was no significant difference of the DNAm between different genotypes (GG, AG, AA) among normal samples, but the DNAm was correlated with the genotypes in RA samples. We observed 0.0168 reduction of the DNAm level at cg00475509 with per allele addition of the rs10796216‐A (Fig. 3A). The DNAm was lower in RA patients (mean value is 0.4536) than that in the normal sample (with a mean of 0.4654), and the *P* value of *T* test was 4.9064e‐4. The result indicated that alteration of DNAm at cg00475509 conferred by allele rs10796216‐A may be associated with the high risk of RA. The rs10796216‐A allele on *FAM107B* may be related to the occurrence and development of the RA process by affecting the DNAm at cg00475509 on *ZIC1* (Zic family member 1).

Located on chromosome 3, *ZIC1* is a known meningeal identity gene. Studies have shown that *ZIC1* promoter methylation was related to the etiology of postmenopausal osteoporosis [16], and patients with RA are at high risk of osteoporosis [17, 18, 19, 20]. Our findings suggest that *ZIC1* may be associated with RA susceptibility, and deserve further in‐depth research.

### Strong regulatory relationships between rs6546473 and cg13358873

Another regulatory relationship strongly associated with RA that was only detected in RA samples was the rs6546473‐cg13358873 pair, which is a cis meQTL located at gene *ANTXR1* on chromosome 2 (Fig. 2). The mean value of DNAm at cg13358873 is 0.4210 among normal samples and 0.3703 among RA patients, with a significant difference of 0.0507 (*P* = 2.0407e‐28) (Fig. 3B). Additionally, the DNAm at cg13358873 decreased by 0.0365 for each addition of allele A at rs6546473, and the lower DNAm at cg13358873 conferred by the allele of rs6546473‐A was significantly associated with the high risk of RA.

*ANTXR1* is a protein coding gene located on chromosome 2, encoding the type 1 cross‐membrane protein, which is a tumor endothelial logo related to colorectal cancer. The GO term related to *ANTXR1* gene includes the combination of receptor activity and musclebin wire. *ANTXR1* plays a vital role in cell attachment and migration, mediating the reorganization of the muscle protein cell skeleton and promoting the diffusion of cells. At present, most of the research on *ANTXR1* is related to colorectal cancer. But the relationship of *ANTXR1* and the pathogenesis of RA has not yet been studied. Our results suggested that the genetic variation at gene *ANTXR1* may be related to the RA pathogenesis by the alteration of the DNA methylation level, which is worthy of in‐depth study.

### Other regulatory relationships unique in RA

There were many other regulatory relationships that exist only in RA samples (Table S1). For example, the *EPS8L3* gene has the largest number of meQTLs, a total of three meQTLs governed by rs3818562 located at a gene region. The DNA methylation sites involved in these meQTLs were all located in *GSTM3*, *GSTM5*, and *RP4‐735C1.4* genes. Both *GSTM3* and *GSTM5* are mu‐like glutathione *S*‐transferases, which plays an important role in the detoxification of electrophilic compounds by binding to glutathione. Studies have shown that they are highly polymorphic, and genetic variation in these genes can alter the susceptibility to carcinogens and toxins and affect individual efficacy of certain drugs and toxicities. It has been reported that the genotype of some glutathione transferase maybe a genetic factor that determines RA susceptibility [21], such as *GSTM1* homozygous null genotype [22], and *GSTT1*‐0 genotype [21, 23]. Our findings suggest that genotypes of SNP rs3818562 at the *EPS8L3* gene may be associated with individual DNA methylation level changes of CpGs at *GSTM3*, and *GSTM5*. These changes may be related to the inflammation induced by RA or the effect of the drug used.

### Regulatory relationships unique in normal samples

Some regulatory relationships were significant in normal samples but were missing in RA patients, indicating that the absence of regulatory relationships may be related to the development of disease processes. There are 12 pairs of such meQTLs identified, composed of 12 unique CpG sites that regulated by seven unique SNPs. *RP11‐718O11.1* covers most of the meQTLs, with a total of four (Fig. 2).

Three of the meQTLs detected were unique in normal samples and were focused on in our study. The first one is a trans meQTL composed of the cg06321045 located at gene *PSORS1C1* on chromosome 6 that is regulated by rs951295 located at gene *RP11‐718O11.1* on chromosome 15 (Fig. 3C). The DNAm at cg06321045 increased by 0.0351 with addition of allele rs951295‐A. And the DNAm is higher in normal samples than RA patients (mean difference is 0.0162, *P* = 0.0094). The second one is a cis meQTL composed of cg12086421 located at the intergenic region on chromosome 6 that is regulated by rs9363764 located at intergenic region on chromosome 6 (Table S1). The DNA methylation of cg12086421 decreased by 0.0413 for each additional allele rs9363764‐A, and the mean value of DNAm was 0.0162 lower in normal samples than in RA patients. The third one is the rs951295‐cg24735489 pair, which is also a trans regulatory relationship between chromosome 6 and chromosome 15 (Fig. 3D). The mean DNAm in normal samples was 0.3486, which is 0.0110 higher than that in RA patients (*P* = 0.0236). There was a 0.0279 increase for each addition of allele rs951295‐A, indicating that the higher DNAm is affected by the genotype of rs951295. The DNA methylation site cg24735489 located at the *PSORS1C1* gene near the major histocompatibility complex (*MHC*) class I region, and one of the drugs for *PSORS1C1*, etanercept, was used for the treatment of a variety of inflammatory conditions including RA [24]. We speculate that the higher DNAm at cg24735489 conferred by rs951295‐A on the *PSORS1C1* gene may be associated with the occurrence and development of RA or the effect of drugs used for treatment.

### Function annotation of meQTL sites

The identified CpG sites influenced by genetic variants are enriched in the KEGG pathways related to glutathione metabolism, Chemical carcinogenesis—DNA adducts, drug metabolism—cytochrome P450, platinum drug resistance, metabolism of xenobiotics by cytochrome P450, drug metabolism—other enzymes, fluid shear stress and atherosclerosis, hepatocellular carcinoma, chemical carcinogenesis—receptor activation and chemical carcinogenesis—reactive oxygen species. There were four genes involved in these functions: *GSTM5* (Glutathione *S*‐Transferase Mu 5), *GSTM3* (Glutathione *S*‐Transferase Mu 3), *GSTM2* (Glutathione *S*‐Transferase Mu 2), and *GSTM1* (Glutathione *S*‐Transferase Mu 1) (Table 1).

**Table 1:** KEGG pathways and GO terms for CpG sites of meQTLs.

ID	Description	*P* value	*q* Value	Gene symbol
hsa00480	Glutathione metabolism	1.44E–06	9.90E–06	GSTM5/GSTM3/GSTM2/GSTM1
hsa05204	Chemical carcinogenesis—DNA adducts	3.12E–06	9.90E–06	GSTM5/GSTM3/GSTM2/GSTM1
hsa00982	Drug metabolism—cytochrome P450	3.70E–06	9.90E–06	GSTM5/GSTM3/GSTM2/GSTM1
hsa01524	Platinum drug resistance	3.91E–06	9.90E–06	GSTM5/GSTM3/GSTM2/GSTM1
hsa00980	Metabolism of xenobiotics by cytochrome P450	5.10E–06	9.90E–06	GSTM5/GSTM3/GSTM2/GSTM1
hsa00983	Drug metabolism—other enzymes	5.65E–06	9.90E–06	GSTM5/GSTM3/GSTM2/GSTM1
hsa05418	Fluid shear stress and atherosclerosis	5.04E–05	7.59E–05	GSTM5/GSTM3/GSTM2/GSTM1
hsa05225	Hepatocellular carcinoma	0.0001	0.0001	GSTM5/GSTM3/GSTM2/GSTM1
hsa05207	Chemical carcinogenesis—receptor activation	0.0003	0.0003	GSTM5/GSTM3/GSTM2/GSTM1
hsa05208	Chemical carcinogenesis—reactive oxygen species	0.0003	0.0003	GSTM5/GSTM3/GSTM2/GSTM1

Moreover, function annotation analysis was performed for the meQTLs significant only in RA samples or normal samples (Tables S2 and S3). There are two pathways related to drug metabolism annotated for the CpG sites of meQTLs uniquely in RA samples, indicating that the meQTL effect identified may be an effect of the inflammation induced by RA or that of the medications being used.

## Discussion

In this study, meQTL analysis was performed on DNA methylation data and SNP genotype data from 689 Swedish (354 RA patients, 335 normal samples). We scanned linear relationships between –DNA methylation level of 485,512 CpG and genotypes of 62 SNP sites, and finally identified 65 significantly related regulatory relationship pairs with meQTL effects, of which 51 were cis meQTLs. Among these meQTLs, 21 regulatory relationship pairs were significantly detected only in RA samples, and 12 were only significantly detected in normal samples, indicating the change of –regulatory relationship. The function annotation results of CpG sites of meQTLs were significant only in RA samples, including two pathways related to drug metabolism. We suspect that these changes may have contributed to the development of RA, or that they may be triggered by the inflammation induced by RA or the effects of the medications being used. Either way, these changes are highly related to the mechanism of RA and deserve further investigation.

Considering this feature, they can be subsequently applied to RA risk assessment studies as risk markers. For example, the rs6546473‐cg13358873 pair, a cis‐regulatory relationship pair located on the *ANTXR1* gene, was only significant in RA samples. With each additional allele G of rs6546473, the DNA methylation level of cg13358873 was increased by 0.0365 in RA samples. The rs6546473‐G allele was supposed as a protective allele for the risk of RA and was significantly associated with hypomethylation of cg13358873 (*P* = 2.0407e‐28) in our study. In addition, meQTLs with opposite regulatory directions in the normal samples and RA samples can also be considered as RA risk markers in future studies. But in the present study, no such relationship was found.

Finally, it is worth noting that there are some SNP‐CpG pairs that have obvious meQTL effects visible from the boxplots, but have not been identified, such as –rs10033147‐cg10279535 relationship pair and rs10796216‐cg25622597 relationship pair (Fig. S1). The reason behind this is that they did not pass the significance threshold after FDR correction. These regulatory relationships are also well worth further study.

## Conflict of interest

The authors declare no conflict of interest.

## Author contributions

JX, YJ, and MZ conceived and contributed to the work. GT, CS, and HL performed research, drafted, and modified the article. All authors contributed to discussing and revising the article.

## Supporting information


**Fig. S1.** Boxplot of (A) rs10796216‐cg25622597 and (B) rs10033147‐cg10279535 relationship pairs.
**Table S1.** Summary information of 64 meQTLs identified with FDR <0.05.
**Table S2.** KEGG pathways and GO Terms annotation for CpG sites of meQTLs only in normal samples.
**Table S3.** KEGG pathways and GO Terms annotation for CpG sites of meQTLs only in RA samples.

## Data Availability

The data that support the findings of this study are available in –GEO (Gene Expression Omnibus) database at https://www.ncbi.nlm.nih.gov/geo/, number GSE42861.
