# Supplementary material for: Identification of novel meQTLs strongly associated with rheumatoid arthritis by large‐scale epigenome‐wide analysis
Source: FEBS Open Bio. 2022 Nov 19;12(12):2227–35. doi: 10.1002/2211-5463.13517 (PMC9714356; doi:10.1002/2211-5463.13517)
Supplement: Supplementary file 1 — Fig. S1. Boxplot of (A) rs10796216‐cg25622597 and (B) rs10033147‐cg10279535 relationship pairs. Table S1. Summary information of 64 meQTLs identified with FDR <0.05. Table S2. KEGG pathways and GO Terms annotation for CpG sites of meQTLs only in normal samples. Table S3. KEGG pathways and GO Terms annotation for CpG sites of meQTLs only in RA samples. [file FEB4-12-2227-s001.pdf]

Supplementary Table S1. Summary information of 65 meQTLs identified with FDR<0.05.

cg_rs	sig_type	qtl_type	coeff.nor	Pvalue.nor	coeff.ra	Pvalue.ra	rs.CHR	rs.gene	cg.CHR	cg.gene
cg00168967_rs3818562	nor	trans	0.0201	1.36E-06	0.0031	4.64E-01	1	EPS8L3	8	ASPH
cg00297950_rs3818562	both	cis	0.0229	7.63E-07	0.0352	9.32E-25	1	EPS8L3	1	GSTM5|RP4-735C1.4|GSTM3
cg25027501_rs3818562	RA	cis	0.0094	3.20E-04	0.0154	1.43E-16	1	EPS8L3	1	GSTM5|RP4-735C1.4|GSTM3
cg01749753_rs2032088	both	cis	0.0473	1.74E-29	0.0315	2.23E-17	21	TTC3	21	DSCR9
cg02592586_rs1510480	both	cis	-0.0309	1.81E-18	-0.0291	1.50E-19	2		2	
cg20991152_rs2804694	RA	cis	-0.0386	4.54E-04	-0.0790	5.55E-12	1		1	
cg05376982_rs3818562	both	cis	-0.0765	5.18E-12	-0.1122	1.22E-23	1	EPS8L3	1	
cg05472380_rs2959823	nor	long_cis	-0.1075	1.02E-09	-0.0559	3.53E-04	15	C15orf27	15	PEAK1
cg06321045_rs951295	nor	trans	0.0351	2.89E-07	-0.0025	6.82E-01	15	RP11-718O11.1	6	PSORS1C1
cg06512774_rs2032088	nor	cis	0.0200	5.45E-09	0.0136	1.27E-06	21	TTC3	21	TTC3
cg06527936_rs951295	both	cis	-0.0498	2.50E-17	-0.0575	8.93E-16	15	RP11-718O11.1	15	
cg07014020_rs348937	both	cis	-0.0308	1.78E-09	-0.0368	2.40E-09	5		5	MCC|CTD-2201G3.1
cg07243138_rs1945975	both	cis	-0.0296	4.32E-23	-0.0211	5.64E-14	11	RP11-94P11.4	11	RP11-94P11.4
cg07319199_rs6546473	both	cis	-0.2015	4.18E-44	-0.1890	3.00E-39	2	ANTXR1	2	ANTXR1
cg13358873_rs6546473	RA	cis	0.0145	1.25E-02	0.0365	5.91E-11	2	ANTXR1	2	ANTXR1
cg09456493_rs10936224	RA	cis	0.0127	2.92E-07	0.0152	1.39E-10	3	NMD3	3	PPM1L
cg25520068_rs3818562	RA	cis	-0.0385	4.17E-04	-0.0729	2.48E-10	1	EPS8L3	1	
cg09050820_rs845016	both	trans	-0.1023	4.71E-30	-0.1044	7.09E-38	21		6	TCP10L2
cg09164898_rs951295	nor	trans	0.0160	4.88E-07	-0.0026	2.97E-01	15	RP11-718O11.1	1	EDARADD
cg10321404_rs11249206	RA	cis	0.0326	1.06E-04	0.0499	2.49E-10	1	RUNX3	1	RP11-84D1.2
cg09694986_rs6991394	both	cis	-0.1156	2.07E-32	-0.1410	1.84E-30	8	RP11-713M15.1	8	SNTB1|RP11-713M15.1
cg15007548_rs264581	RA	cis	0.0199	2.62E-04	0.0342	7.94E-10	2	TANC1	2	TANC1
cg04134399_rs6426327	RA	cis	0.0199	8.15E-03	0.0427	1.09E-09	1	SMYD3	1	SMYD3
cg10807101_rs3818562	both	cis	0.0443	3.80E-16	0.0541	4.05E-25	1	EPS8L3	1	GSTM5|RP4-735C1.4|GSTM3

cg_rs	sig_type	qtl_type	coeff.nor	Pvalue.nor	coeff.ra	Pvalue.ra	rs.CHR	rs.gene	cg.CHR	cg.gene
cg10950028_rs3818562	both	cis	-0.0656	1.27E-10	-0.1044	7.28E-22	1	EPS8L3	1	GSTM2|GSTM1
cg11680055_rs3818562	both	cis	-0.0735	1.13E-11	-0.1008	1.21E-20	1	EPS8L3	1	GSTM2
cg11921048_rs3818562	nor	trans	0.0103	1.26E-06	0.0025	2.01E-01	1	EPS8L3	3	
cg12086421_rs9363764	nor	cis	-0.0413	1.53E-12	-0.0191	3.52E-05	6		6	
cg12800200_rs6546473	both	cis	-0.0213	6.79E-15	-0.0226	3.98E-24	2	ANTXR1	2	ANTXR1
cg12858902_rs3818562	both	cis	-0.0507	3.96E-10	-0.0729	5.35E-19	1	EPS8L3	1	
cg22117498_rs7660805	RA	cis	0.0131	2.81E-05	0.0204	4.39E-09	4		4	
cg14377951_rs3818562	both	cis	-0.0394	1.11E-09	-0.0537	5.87E-17	1	EPS8L3	1	GSTM5
cg07416590_rs715359	RA	cis	0.0083	1.49E-06	0.0097	7.64E-09	1	RP1-35C21.2	1	BRINP2
cg15089219_rs951295	nor	trans	0.0157	1.46E-07	-0.0019	4.85E-01	15	RP11-718O11.1	2	C1QL2
cg15201175_rs6426327	nor	trans	-0.0451	9.44E-08	0.0004	9.69E-01	1	SMYD3	5	SEMA6A
cg15259449_rs7660805	both	cis	-0.0102	1.54E-09	-0.0099	2.31E-08	4		4	
cg16416158_rs213028	both	cis	0.0337	6.02E-26	0.0435	1.03E-49	1	ECE1	1	ECE1
cg18450420_rs10796216	both	cis	0.0487	1.22E-17	0.0508	4.08E-19	10	FAM107B	10	FAM107B|RP11-7C6.1
cg19207856_rs2032088	both	cis	0.0053	2.34E-12	0.0035	1.60E-07	21	TTC3	21	PIGP
cg19733633_rs348937	nor	cis	-0.0283	9.29E-09	-0.0259	2.37E-06	5		5	
cg19910937_rs2125573	both	cis	-0.0180	2.44E-14	-0.0152	8.30E-11	4	INTU	4	LARP1B
cg08949101_rs845016	RA	cis	-0.0090	4.65E-03	-0.0161	9.68E-09	21		21	TCP10L|AP000275.65
cg20803293_rs3818562	both	cis	-0.0815	1.51E-12	-0.1118	8.38E-22	1	EPS8L3	1	
cg20857253_rs845016	both	cis	0.0394	9.32E-13	0.0321	6.92E-09	21		21	TCP10L|AP000275.65
cg24312680_rs2125573	RA	cis	0.0362	1.79E-06	0.0420	1.98E-08	4	INTU	4	HSPA4L
cg21832243_rs2032088	both	cis	0.0387	1.75E-16	0.0460	1.16E-22	21	TTC3	21	PIGP
cg25589001_rs10882854	RA	cis	0.0316	7.12E-06	0.0388	4.23E-08	10	LCOR	10	LCOR
cg22864244_rs3818562	both	cis	-0.0648	1.99E-12	-0.0948	1.68E-23	1	EPS8L3	1	GSTM5
cg23645476_rs3818562	both	cis	0.0143	1.07E-07	0.0173	9.26E-17	1	EPS8L3	1	GSTM5|RP4-735C1.4|GSTM3

cg_rs	sig_type	qtl_type	coeff.nor	Pvalue.nor	coeff.ra	Pvalue.ra	rs.CHR	rs.gene	cg.CHR	cg.gene
cg23719124_rs3818562	both	cis	-0.0595	4.93E-10	-0.0845	2.07E-19	1	EPS8L3	1	GSTM5
cg23730617_rs10936224	both	cis	0.0288	5.94E-17	0.0371	2.15E-23	3	NMD3	3	NMD3
cg23947654_rs264581	both	cis	-0.1149	5.15E-15	-0.1175	2.22E-14	2	TANC1	2	TANC1
cg00475509_rs10796216	RA	trans	0.0016	6.55E-01	-0.0168	4.83E-08	10	FAM107B	3	ZIC1
cg20703997_rs3936238	RA	cis	0.0215	4.14E-03	0.0436	8.36E-08	1		1	
cg24467349_rs3818562	both	cis	-0.0810	3.23E-12	-0.1069	1.39E-20	1	EPS8L3	1	
cg24539599_rs348937	RA	cis	0.0150	2.58E-06	0.0171	1.52E-07	5		5	
cg08472449_rs264581	RA	cis	0.0141	9.65E-03	0.0260	1.75E-07	2	TANC1	2	TANC1
cg24735489_rs951295	nor	trans	0.0279	8.48E-08	-0.0016	7.46E-01	15	RP11-718O11.1	6	PSORS1C1
cg24896460_rs3818562	nor	trans	0.0172	1.45E-06	0.0048	2.01E-01	1	EPS8L3	5	ARHGEF28
cg24022357_rs6546473	RA	trans	0.0003	7.89E-01	0.0049	3.52E-07	2	ANTXR1	19	SPPL2B
cg25210835_rs3818562	both	cis	-0.0650	3.61E-10	-0.0911	7.26E-19	1	EPS8L3	1	
cg10462593_rs3818562	RA	trans	0.0051	1.16E-01	0.0155	5.73E-07	1	EPS8L3	15	
cg24506221_rs3818562	RA	cis	-0.0787	1.18E-03	-0.1100	1.22E-06	1	EPS8L3	1	GSTM2
cg25593510_rs3818562	both	cis	-0.0861	1.22E-10	-0.1276	1.56E-21	1	EPS8L3	1	
cg26739410_rs3818562	RA	trans	-0.0023	7.70E-01	-0.0358	1.90E-06	1	EPS8L3	X	FAM47C

Supplementary Table S2. KEGG pathways and GO Terms annotation for CpG sites of meQTLs only in normal samples.

ID	Description	pvalue	qvalue	Gene Symbol
Note: CpG sites of meQTLs only in normal samples has not been enriched in any KEGG pathways or GO Terms.				

Supplementary Table S3. KEGG pathways and GO Terms annotation for CpG sites of meQTLs only in RA samples.

ID	Description	pvalue	qvalue	Gene Symbol
hsa00480	Glutathione metabolism	6.30E-06	6.17E-06	GSTM2/GSTM5/GSTM3
hsa05204	Chemical carcinogenesis - DNA adducts	1.12E-05	6.17E-06	GSTM2/GSTM5/GSTM3
hsa00982	Drug metabolism - cytochrome P450	1.28E-05	6.17E-06	GSTM2/GSTM5/GSTM3
hsa01524	Platinum drug resistance	1.33E-05	6.17E-06	GSTM2/GSTM5/GSTM3
hsa00980	Metabolism of xenobiotics by cytochrome P450	1.63E-05	6.17E-06	GSTM2/GSTM5/GSTM3
hsa00983	Drug metabolism - other enzymes	1.76E-05	6.17E-06	GSTM2/GSTM5/GSTM3
hsa05418	Fluid shear stress and atherosclerosis	9.22E-05	2.77E-05	GSTM2/GSTM5/GSTM3
hsa05225	Hepatocellular carcinoma	1.62E-04	4.26E-05	GSTM2/GSTM5/GSTM3
hsa05207	Chemical carcinogenesis - receptor activation	3.23E-04	7.55E-05	GSTM2/GSTM5/GSTM3
hsa05208	Chemical carcinogenesis - reactive oxygen species	3.75E-04	7.89E-05	GSTM2/GSTM5/GSTM3
GO:0006749	glutathione metabolic process	8.49E-06	1.30E-03	GSTM2/GSTM5/GSTM3
GO:0042178	xenobiotic catabolic process	1.11E-04	6.61E-03	GSTM2/GSTM3
GO:0042537	benzene-containing compound metabolic process	1.30E-04	6.61E-03	GSTM2/GSTM3
GO:0006575	cellular modified amino acid metabolic process	2.03E-04	7.73E-03	GSTM2/GSTM5/GSTM3
GO:0045171	intercellular bridge	1.16E-05	3.65E-04	GSTM2/GSTM5/GSTM3
GO:0004364	glutathione transferase activity	4.10E-07	8.19E-06	GSTM2/GSTM5/GSTM3
GO:0016765	transferase activity, transferring alkyl or aryl (other than methyl) groups	5.07E-06	5.07E-05	GSTM2/GSTM5/GSTM3
GO:0043295	glutathione binding	1.46E-05	8.90E-05	GSTM2/GSTM3
GO:1900750	oligopeptide binding	1.78E-05	8.90E-05	GSTM2/GSTM3
GO:0072341	modified amino acid binding	1.35E-03	5.39E-03	GSTM2/GSTM3
GO:0003714	transcription corepressor activity	5.54E-03	1.79E-02	TCP10L/LCOR
GO:0001161	intronic transcription regulatory region sequence-specific DNA binding	6.55E-03	1.79E-02	SMYD3
GO:1990226	histone methyltransferase binding	7.15E-03	1.79E-02	LCOR

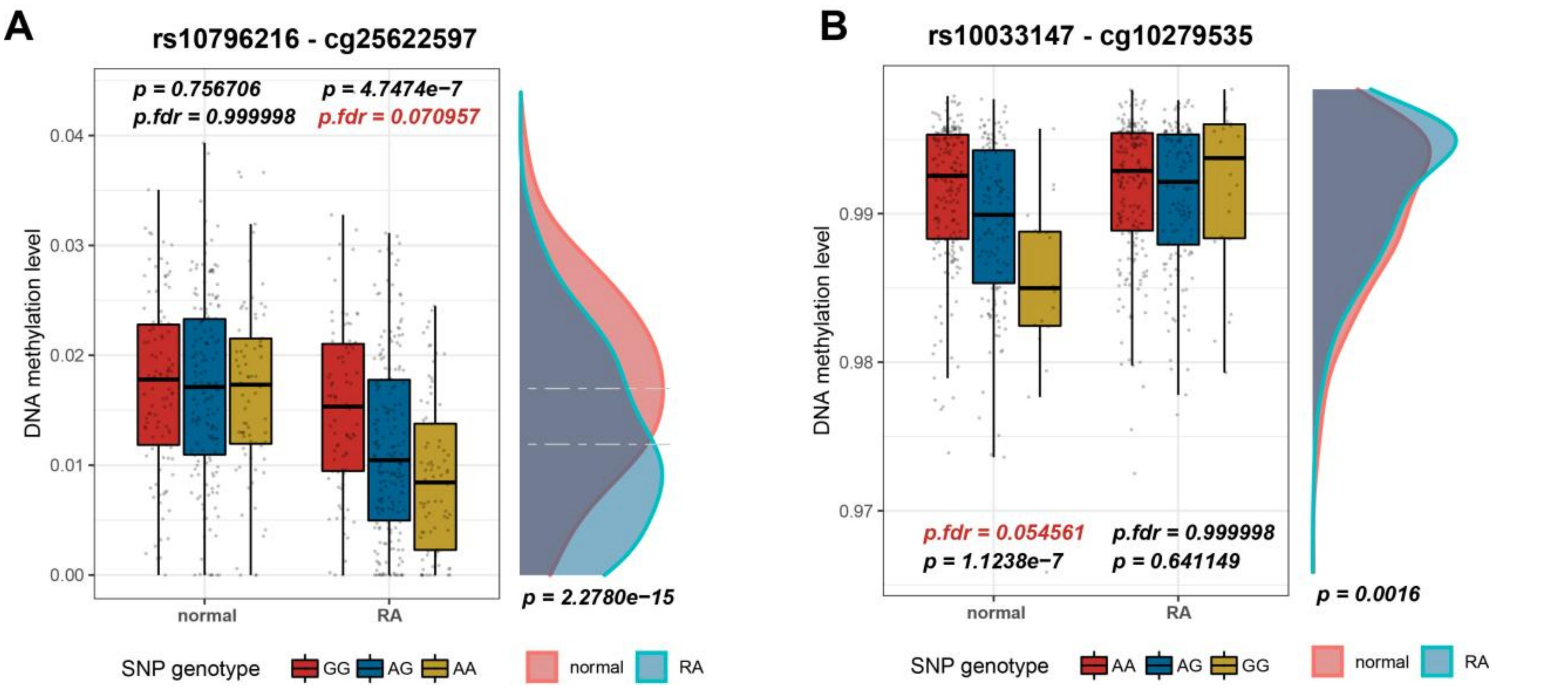

Supplementary Figure S1. Boxplot of (A) rs10796216-cg25622597 and (B) rs10033147-cg10279535 relationship pairs. Two examples of SNP-CpG pairs that have significant regulatory relationship visible in box plots, but were not been identified under FDR<0.05. Relationships between SNP genotypes and DNA methylation level were tested by linear regression with age included as covariate, and the difference of DNA methylation level was identified by t test.
